# Reply: “It is Time for a Universal Nutrition Policy in Very Preterm Neonates during the Neonatal Period? Comment on: Applying Methods for Postnatal Growth Assessment in the Clinical Setting: Evaluation in a Longitudinal Cohort of Very Preterm Infants *Nutrients* 2019, *11*, 2772”

**DOI:** 10.3390/nu12040983

**Published:** 2020-04-02

**Authors:** Montserrat Izquierdo Renau, Victoria Aldecoa-Bilbao, Carla Balcells Esponera, Beatriz del Rey Hurtado de Mendoza, Martin Iriondo Sanz, Isabel Iglesias-Platas

**Affiliations:** 1 Neonatology Department, Hospital Sant Joan de Déu, Institut de Recerca Sant Joan de Déu, Universidad de Barcelona, BCNatal, Esplugues de Llobregat, 08950 Barcelona, Spain; cbalcells@sjdhospitalbarcelona.org (C.B.E.); bdelrey@sjdhospitalbarcelona.org (B.d.R.H.d.M.); miriondo@sjdhospitalbarcelona.org (M.I.S.); iiglesias@sjdhospitalbarcelona.org (I.I.-P.); 2 Neonatology Department, Hospital Clinic, Universidad de Barcelona, BCNatal, 08028 Barcelona, Spain; valdecoa@clinic.cat

We would like to thank Gounaris et al. [1] for their comprehensive analysis of our work and their insightful comments toward a better understanding of preterm growth and nutrition.

Even though improvement of postnatal growth and identification of risk factors was one of our aims, the major focus of our paper was to reflect on how different approaches to analyzing postnatal growth can result into confusing and even inaccurate conclusions. Current reported percentages of EUGR are indeed heterogeneous, and range from around the figure in their referenced paper of 25% [2,3,4,5] to others more in line with our prevalence of 40–60% [6,7,8]. Nevertheless, and this was one of the points we were trying to highlight, interpretation must be cautious, because the definitions of EUGR are different, or applied at different time points, or over populations with different prevalence of IUGR or different gestational ages.

Indeed, the discordance between the paper by the Greek authors and ours might be partially explained by this. Their growth outcome is measured as the percentage of patients with body weight under the 10th percentile at discharge. As discussed in our manuscript, we favor the use of *z*-score difference as classifying growth outcomes by “a centile cut-off point to define growth restriction implies that the presence of IUGR and the *z*-score of BW will have a huge impact in the final categorization”. The prevalence of SGA/IUGR was double in our patients (7.4% vs. 13.7%) and, as we and others have described [6,9,10], this will have a major impact on postnatal growth. Patients in our sample are also more immature, with a gestational age at birth about one week lower (BPD 26.4 ± 1.8 weeks non-BPD 29.9 ± 1.7 weeks). Additionally, Panagiotounakou et al. set a weight limit of 1500 g in their inclusion criteria. This corresponds to approximately the 50th percentile of weight at around 30 weeks in the Fenton growth charts, and means that, over this gestational age and up to 32 weeks, only newborns with a birth weight under the mean (or a *z*-score below 0) for their gestational age were included. Loss of *z*-score at discharge shows a major inverse correlation with the BW *z*-score, and this is also very likely to have influenced the differences in results.

We would like to state that we are firm believers of the possibility of improving postnatal preterm growth, and that this will start with the identification of risk factors like gestational age or respiratory illness [11,12], even if these are unmodifiable. The influence of initial weight loss, on the other hand, is an important finding and highlights the relevance of an early start to growth monitoring and support [13]. Our restrictive approach to fluid provision might play a role in EUGR, although evidence in this area of neonatal medicine is low and quite old, it does not seem to favor liberal fluid intake, which might increase the risk of morbidity and mortality [14,15,16]. However, we disagree with the appreciation that VPI in our unit had a “high percentage of initial weight loss”, with an average of 8.3% ± 4.6%, which is lower than reported in this population [17,18].

Regarding enteral nutritional policies, the main difference is the supplementation of mother’s milk with formula in the Greek neonatal unit. Human milk has non-nutritional advantages and protects preterm babies from NEC [19], feeding intolerance [20,21], bronchopulmonary dysplasia (BPD) [22] and LOS [23] and might even support better long-term neurodevelopmental and cardiovascular outcomes [24,25]. International scientific societies recommend the use of donor milk as a complement to own mother’s milk (OMM) when the amount of the latter does not cover the volume requirement of the preterm infant [26], even if somatic growth might be slower, which is not always the case [27,28]. We use routine human milk fortification for both OMM and DHM as recommended by scientific societies [29] when 100 mL/kg/day of HM feeds is obtained. Our sample has an average time to reach full enteral feeds of 13 days, with an average amount of milk of 130 mL/kg on the first day without parenteral nutrition, so usually; fortification of milk was started after the first week of life. In any case, 73% of extremely preterm infants (≤28 weeks) in our study were exclusively fed own mother’s milk for the first 28 days of life, and only 7% were receiving solely donor milk.

Although enteral supply in our cohort might seem somewhat low, all analyses were undertaken on actual intakes, rather than prescription. Upper limits recommended by the World Health Organization [30] and ESPGHAN [31] are around 180–200 mL/kg/day, and that is reflected in our protocol. Nevertheless, these volumes were not met, either because of the low prescription by clinicians [32], or due to feeding intolerance, a major problem in preterm enteral nutrition, with up to 40% of infants receiving less milk than initially intended [4]. Incidentally, this seems to be even more of a challenge in the smallest preterm infants and those on non-invasive respiratory support, who tend to develop “CPAP belly syndrome” [33]. Actual feeding volumes of the Greek cohort would have been particularly informative for interpretation.

In conclusion, we have tried to emphasize that improvement of preterm postnatal growth outcomes will first require the standardizations of methods for evaluating and reporting growth. Unexpectedly, nutritional factors had a small impact in growth outcomes in our cohort and we hypothesize that this might indicate that the sickest infants sustain an increased energy expenditure and metabolic interference due to illness and inflammation. Although we do agree that the key for improvement in our unit will have to include optimization of enteral feeding [34], our opinion is that time calls for a more individualized than universal approach, and this will require the development of biochemical and body composition markers as well as the assessment of long term outcomes.
